# Elective Laparoscopic Resection Following Transarterial Embolization and Anticoagulation for a Giant Hepatocellular Adenoma with Hemorrhage and Venous Thrombosis: A Case Report

**DOI:** 10.70352/scrj.cr.26-0311

**Published:** 2026-10-09

**Authors:** Arisa Koshimoto, Hajime Matsushima, Ayaka Kinoshita, Takashi Hamada, Kazushige Migita, Yuki Nunoshita, Shun Nakamura, Fumika Kamehama, Shinichiro Ogawa, Shintaro Ryu, Hajime Imamura, Takanobu Hara, Masato Fujiyoshi, Tomohiko Adachi, Shinji Okano, Susumu Eguchi

**Affiliations:** 1 Department of Surgery, Division of Digestive Surgery and Transplantation, Nagasaki University Graduate School of Biomedical Sciences, Nagasaki, Nagasaki, Japan; 2 Department of Pathology, Nagasaki University Graduate School of Biomedical Sciences, Nagasaki, Nagasaki, Japan

**Keywords:** hepatocellular adenoma, laparoscopic, transarterial embolization, hepatectomy

## Abstract

**INTRODUCTION:**

Hepatocellular adenoma (HCA) is a rare benign liver tumor that can be complicated by hemorrhage, whereas concomitant major venous thrombosis is uncommon.

**CASE PRESENTATION:**

A 25-year-old woman presented with epigastric pain and nausea. Contrast-enhanced CT showed a 17-cm liver tumor involving the anterior and medial segments, which suggested HCA with intratumoral hemorrhage. She initially underwent transarterial embolization (TAE) for hemostasis at a previous hospital. Subsequent CT showed thrombi in the inferior vena cava and pulmonary artery, and she was referred to our institution. On the basis of her stable condition and the presumed benign nature of the tumor, anticoagulation therapy was initiated to treat the thromboses, and elective surgery was planned. After 8 months, the thrombi had resolved, and the tumor had decreased in size. Laparoscopic central bisectionectomy was then successfully performed. The postoperative course was uneventful, and the patient was discharged on POD 8.

**CONCLUSIONS:**

In patients with a large HCA complicated by hemorrhage and venous thromboses, a staged approach consisting of TAE and anticoagulation may enable safe and minimally invasive elective laparoscopic hepatectomy.

## Abbreviations

AFP
: alpha-fetoprotein
ALT
: alanine aminotransferase
AST
: aspartate aminotransferase
EOB-MRI
: ethoxybenzyl MRI
HCA
: hepatocellular adenoma
HCC
: hepatocellular carcinoma
INR
: international normalized ratio
IVC
: inferior vena cava
PIVKA-II
: protein induced by vitamin K absence-II
TAE
: transarterial embolization

## INTRODUCTION

HCA is a rare benign liver tumor that predominantly occurs in young women. The development of HCA has been reported to be associated with exogenous hormonal exposure, particularly the use of oral contraceptives, as well as endogenous hormonal influences and metabolic factors.^1–3)^ Most cases of HCA are asymptomatic and are often detected incidentally. Therefore, HCA may grow to a large size before its diagnosis.^4,5)^ Importantly, HCA can be complicated by intratumoral hemorrhage or malignant transformation, both of which occasionally lead to life-threatening clinical conditions.^6)^ The management of HCA is often complex and requires a tailored approach based on tumor characteristics, molecular subtypes, and individual patient-specific factors.^7)^ Acute complications such as hemorrhage require prompt intervention, and transarterial TAE has been widely accepted as an initial treatment option in such settings.^8,9)^ Furthermore, the presence of concomitant conditions or complications can further complicate treatment strategies and necessitate a multidisciplinary approach to optimize individualized management.^9)^ Although HCA is rare, it has been reported to enlarge and compress the IVC, potentially leading to impaired venous flow and thrombus formation.^10)^

We report a rare case of giant HCA complicated by intratumoral hemorrhage and concomitant IVC thrombosis, in which anticoagulation therapy was prioritized, followed by successful elective laparoscopic liver resection. We describe the clinical course of our patient and discuss the management strategy for this condition.

## CASE PRESENTATION

A 25-year-old woman with no notable medical history and no history of oral contraceptive or other hormonal medication use presented with epigastric discomfort and nausea. She visited a local clinic because of worsening abdominal pain. Non-contrast CT showed a large liver tumor, and she was referred to a previous hospital for further evaluation. Physical examination revealed a BMI of 21 kg/m^2^. The abdomen was flat and soft, without tenderness, palpable mass, or hepatic percussion tenderness. Laboratory examination showed hemoglobin of 9.1 g/dL, AST 104 U/L, ALT 61 U/L, albumin 3.7 g/dL, total bilirubin 0.7 mg/dL, and INR 1.01. The patient had preserved liver function, with a Child–Pugh score of 5 (class A). With regard to tumor markers, serum AFP level was 2.0 ng/mL, PIVKA-II level was 61.5 mAU/mL, and carcinoembryonic antigen and carbohydrate antigen 19-9 levels were <1.7 ng/mL and <2.1 U/mL, respectively. Contrast-enhanced CT showed a 17-cm hypervascular liver tumor involving the anterior and medial segments. MRI showed heterogeneous intensity on T1-weighted imaging and marked high-intensity areas on T2-weighted imaging, which suggested a hematoma and necrosis. EOB-MRI demonstrated gadoxetic acid uptake within the solid component of the hepatic tumor in the hepatobiliary phase, suggesting a β-catenin–activated HCA (**Fig. 1**). During hospitalization, progressive anemia was observed, and contrast-enhanced CT showed intratumoral hemorrhage. TAE was performed initially to obtain hemostasis, and thereafter, epigastric discomfort was resolved.

**Fig. 1:**
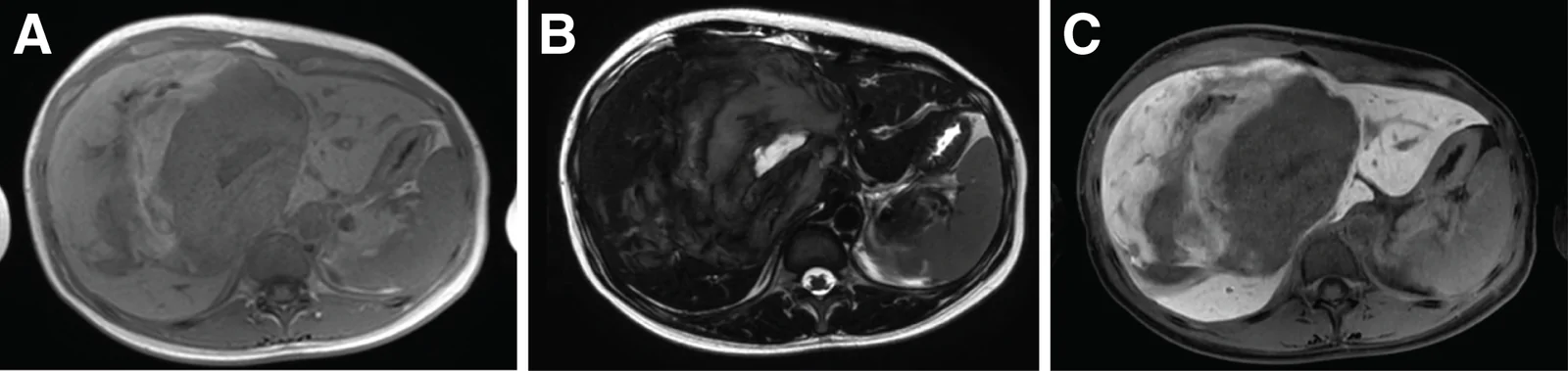
Preoperative contrast-enhanced CT scan. CT shows a 17-cm tumor in segment 4, with heterogeneous arterial enhancement of the solid component (**A**) and isoenhancement relative to the surrounding liver in the portal venous (**B**) and equilibrium phases (**C**).

She was referred to our hospital for further treatment, including surgery. At that time, contrast-enhanced CT showed heterogeneous enhancement of the tumor in the arterial phase and isoenhancement in the portal and equilibrium phases (**Fig. 2**). There was no washout in the delayed phase, and the tumor contained various components, including fat, hemorrhage, and necrosis. Additionally, thrombi were observed in the IVC and left pulmonary artery (**Fig. 3**). Given the absence of a previous history of thrombotic events and the rapid development of the IVC thrombus within approximately 3 weeks in the setting of marked IVC compression by the giant hepatic tumor, venous stasis secondary to mechanical compression was considered the most likely cause of thrombus formation. Therefore, thrombophilia screening was not performed. Given that the EOB-MRI findings were highly suggestive of HCA and that surgical resection at this stage was considered to have a high risk of thromboembolism and required preparations, such as extracorporeal membrane oxygenation or placement of a venous filter, anticoagulation therapy was initiated before surgery.

**Fig. 2:**
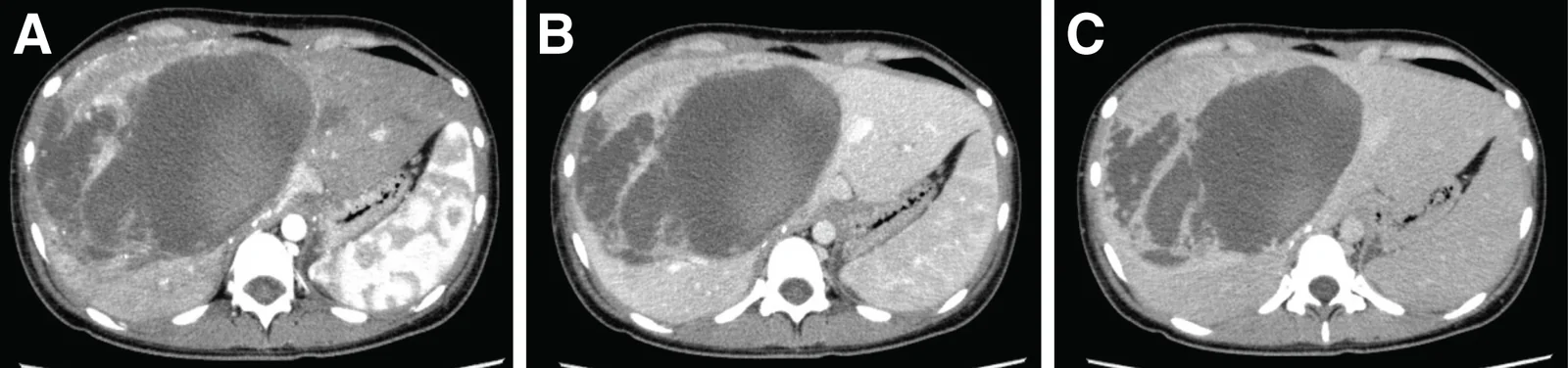
Preoperative MRI, including gadoxetic acid-enhanced imaging. (**A**) T1-weighted imaging shows heterogeneous signal intensity. (**B**) T2-weighted imaging shows focal high-intensity areas suggestive of hematoma and necrosis. (**C**) In the hepatobiliary phase, partial uptake was observed.

**Fig. 3:**
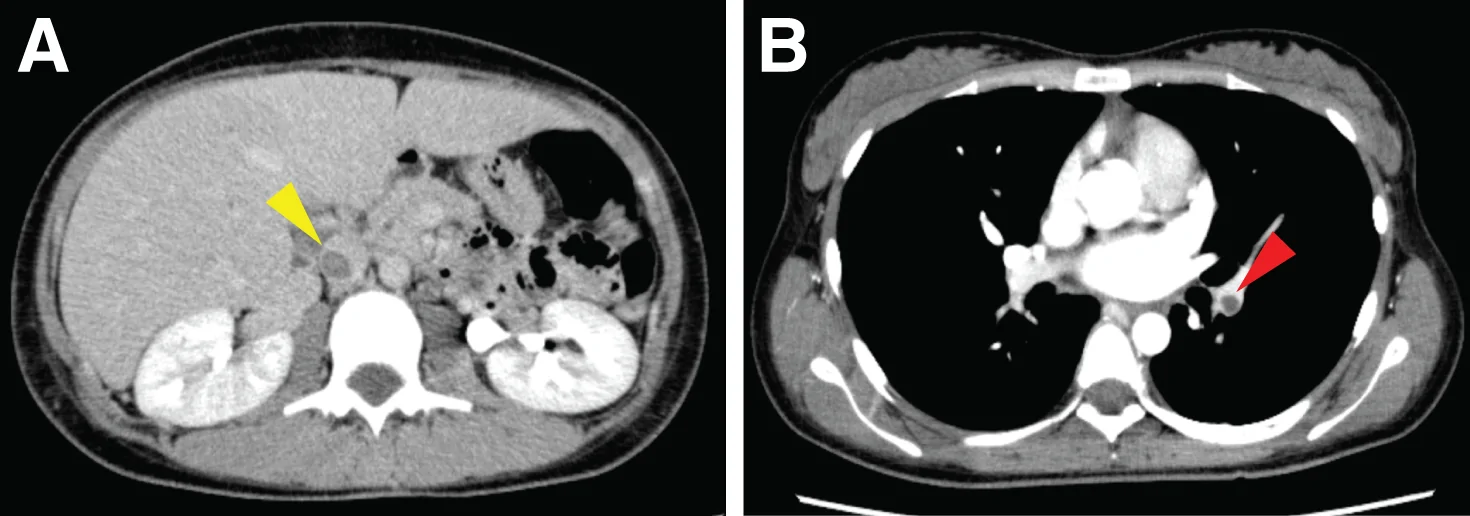
Preoperative contrast-enhanced CT images. (**A**) In the equilibrium phase, a thrombus was observed in the inferior vena cava (yellow arrowhead). (**B**) In the arterial phase, a thrombus was observed in the left pulmonary artery (red arrowhead).

After 8 months of anticoagulation therapy, the size of the tumor decreased from 17.0 to 12.3 cm in diameter. Furthermore, tumor shrinkage was accompanied by improvement of the IVC compression, with complete resolution of the thrombi. Serial CT images obtained from the initial presentation through the period of anticoagulation therapy are shown in **Fig. 4**. At 8 months of anticoagulation therapy, the serum AFP and PIVKA-II levels were 1.0 ng/mL and 47 mAU/mL, respectively, and had not increased from the levels observed at the initial presentation. In addition, liver function was well preserved, with AST 13 U/L, ALT 8 U/L, albumin 4.2 g/dL, total bilirubin 0.5 mg/dL, and INR of 1.06. The Child–Pugh score was 5 (class A), indicating preserved hepatic functional reserve. Laparoscopic central bisectionectomy was performed electively. At the hepatic hilum, the Glissonean pedicle of the anterior section was isolated, and a test clamp was applied, and the demarcation between the anterior and posterior sections was confirmed using indocyanine green imaging (**Fig. 5**). Parenchymal transection was initiated on the right side of the umbilical portion of the portal vein, and the Glissonean pedicle of the medial section was dissected in a caudal-to-cranial direction. Subsequently, parenchymal transection along the boundary between the anterior and posterior sections was performed. After adequate space was secured at the hepatic hilum, the Glissonean pedicle of the anterior section was divided using a linear stapler. Transection of liver parenchyma was performed under the Pringle maneuver. The resected specimen was extracted via a Pfannenstiel incision. The operative time was 516 min. Although no major intraoperative bleeding event occurred, persistent oozing from the liver transection surface during parenchymal transection, likely due to marked peritumoral vascular proliferation, resulted in a total blood loss of 1400 g. The resected specimen weighed 862 g. Gross examination revealed a well-circumscribed, encapsulated tumor measuring 125 × 108 × 118 mm, consisting of a solid component and a hematomatous component that was considered to reflect intratumoral hemorrhage. Histopathological examination demonstrated hemorrhage, necrosis, and hemosiderin deposition within the solid component. The lesion was composed of mildly atypical hepatocellular proliferation without definitive histological features of malignancy. Surgical margins were negative. On the basis of the histopathological examination, the tumor was diagnosed as HCA. Immunohistochemical analysis showed diffuse expression of glutamine synthetase, whereas nuclear expression of β-catenin was negative. Based on the overall histopathological and immunohistochemical findings, the tumor was classified as unclassified HCA (**Fig. 6**). The postoperative course was uneventful, and the patient was discharged on POD 8.

**Fig. 4:**
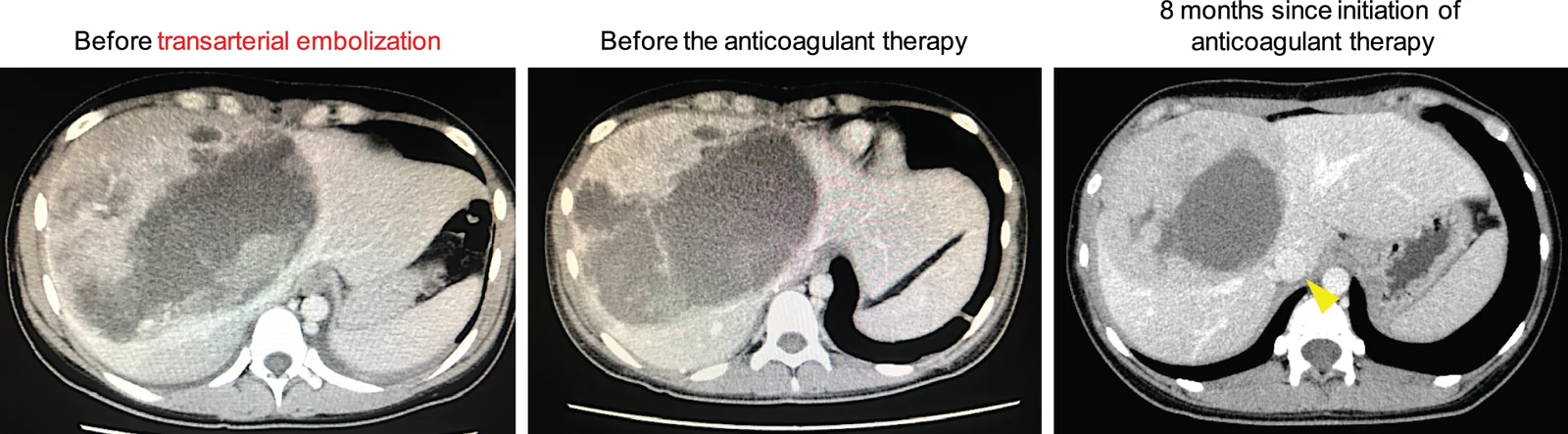
Contrast-enhanced CT scan obtained from the initial presentation through the period of anticoagulation therapy. CT shows reduction of the tumor size to 12.3 cm during 8 months after initiation of anticoagulant therapy. As the tumor decreased in size, the degree of inferior vena cava compression also improved (yellow arrowhead).

**Fig. 5:**
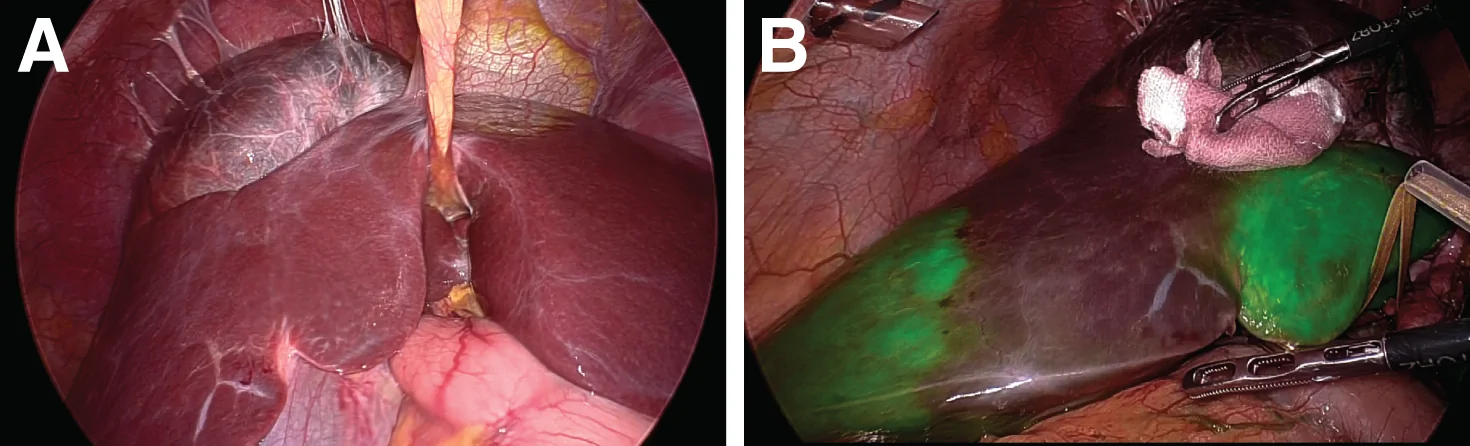
Intraoperative findings. (**A**) The tumor was located in the anterior and medial segments with minimal adhesion to the abdominal wall. (**B**) After isolation of the anterior Glissonean pedicle, the demarcation line along with the boundary between the anterior and posterior sections was confirmed using indocyanine green imaging.

**Fig. 6:**
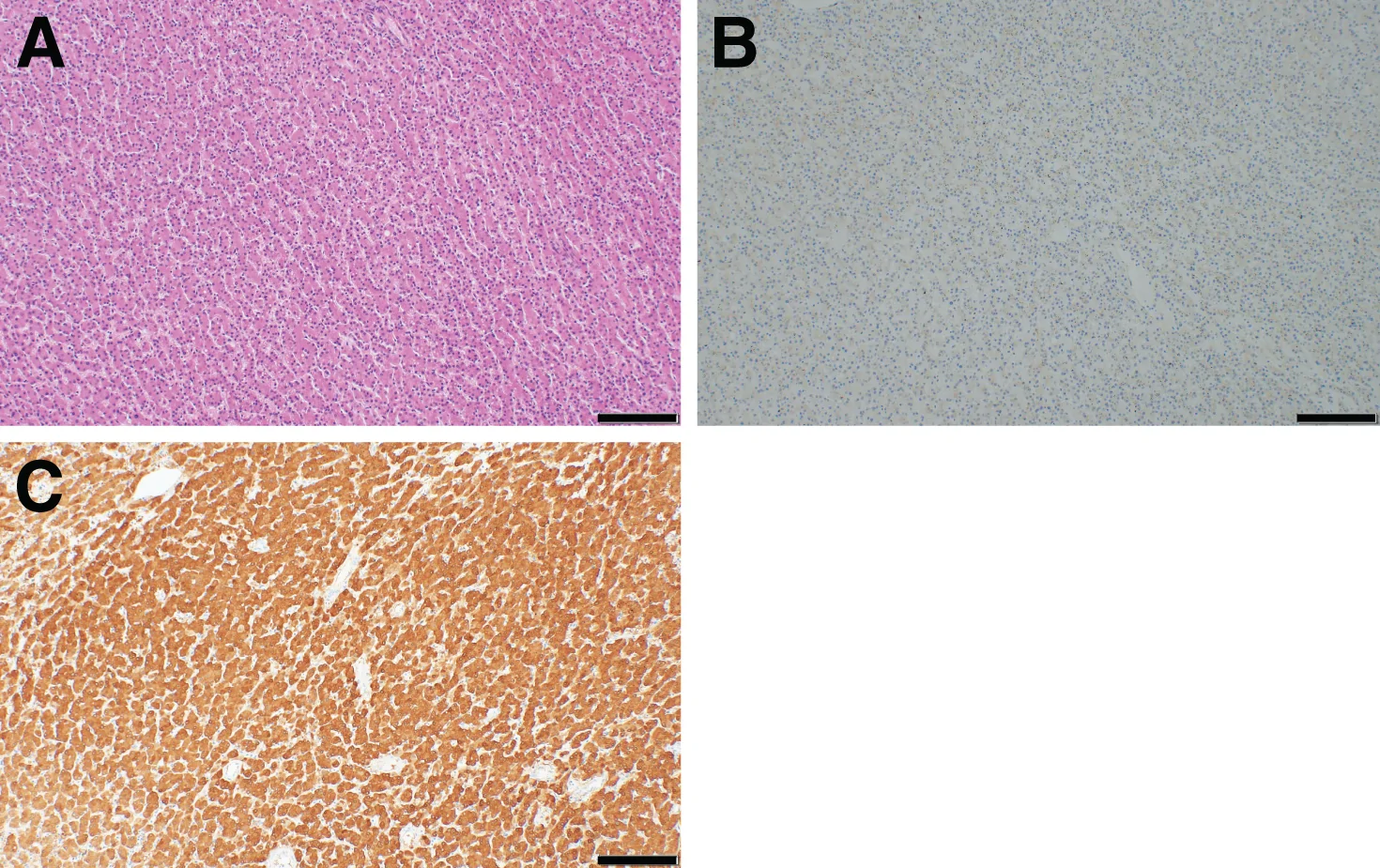
Histopathological and immunohistochemical findings of the resected tumor. (**A**) Hematoxylin and eosin staining (**B**) Immunohistochemical staining for β-catenin (**C**) Immunohistochemical staining for glutamine synthetase. Scale bars, 200 μm.

## DISCUSSION

HCA is a rare benign liver tumor, accounting for approximately 2% of all hepatic neoplasms,^6)^ with an estimated prevalence of 0.001%–0.004% in the general population.^9)^ HCA predominantly occurs in women of reproductive age and is strongly associated with long-term use of oral contraceptives. Additionally, obesity, metabolic syndrome, and genetic predisposition have been reported as risk factors for HCA, reflecting the multifactorial nature of this disease.^1,2)^ Although HCA is often asymptomatic, clinically significant complications include hemorrhage and malignant transformation.^5)^ Hemorrhage has been reported in approximately 25% of HCA cases, with the risk associated with tumor size.^11)^ Furthermore, symptomatic patients are more likely to develop hemorrhagic complications than asymptomatic individuals.^12)^ Malignant transformation is relatively uncommon in women (approximately 4%–5%) but has been described to occur more frequently in men.^13–15)^ This case is noteworthy for several reasons. First, a giant HCA measuring 17 cm developed in the absence of typical risk factors, such as oral contraceptive use or chronic liver disease. Second, the tumor was complicated by intratumoral hemorrhage, which is a potentially life-threatening condition. Third, and most importantly, the patient presented with extensive thrombosis involving the IVC and pulmonary artery. Thromboembolic complications, including pulmonary embolism, have been reported in patients with HCA.^9)^ However, these complications are typically associated with systemic conditions, such as coagulation disorders, inflammatory states, or the prothrombotic effects of oral contraceptives. However, in the present case, venous stasis caused by direct mechanical compression of the IVC by the giant tumor was considered the primary mechanism of the thrombosis. Although a few reports have described IVC compression by a large HCA,^10)^ cases clearly demonstrating this mechanism remain extremely rare, underscoring the uniqueness of this case.

According to the EASL clinical practice guidelines on the management of benign liver tumors, treatment of HCA should be individualized according to patient characteristics and molecular subtype.^16)^ Surgical resection is generally recommended for HCA irrespective of size in male patients and for β-catenin–activated HCA because of the increased risk of malignant transformation. In women, surgical resection should also be considered for HCAs ≥5 cm. In cases complicated by intratumoral hemorrhage, TAE may be performed as the initial treatment, followed by imaging surveillance of the residual tumor to determine the need for subsequent surgical resection. In the present case, the tumor measured 17 cm and was complicated by intratumoral hemorrhage. In addition, the EOB-MRI findings, including uptake within the solid component during the hepatobiliary phase, raised suspicion for β-catenin–activated HCA. Therefore, surgical resection was considered; however, given the presence of intratumoral hemorrhage, TAE was performed as the initial treatment. During subsequent evaluation for surgical resection, the IVC and pulmonary artery thrombosis developed, which was considered to increase the risk of immediate surgery. Anticoagulation therapy was therefore initiated, followed by elective surgical resection after resolution of the thrombus. This strategy minimized the perioperative risk and enabled safe surgical intervention. This case highlights the importance of individualized, multidisciplinary management in HCA with complex complications. Although β-catenin–activated HCA was suspected preoperatively based on the imaging findings, the final pathological diagnosis was unclassified HCA. Current guidelines indicate that reliable differentiation of β-catenin–activated HCA from unclassified HCA and HCC cannot be achieved by imaging alone.^16)^ Thus, the discrepancy between the preoperative imaging-based suspicion and the final pathological subtype in this case underscores the limitations of imaging-based subclassification of HCA. Since excluding the possibility of a well-differentiated HCC is difficult based on the imaging findings alone,^7,17,18)^ the indication for surgical resection should be determined comprehensively, taking into account factors such as patient sex, tumor size, and changes in tumor characteristics over time.

Previously, emergency hepatectomy was commonly performed for the management of hemorrhagic HCA. However, this approach was associated with a high perioperative risk, including mortality rates of 5%–10%.^11,15)^ Currently, a stepwise strategy consisting of initial hemostasis with TAE, followed by elective resection after stabilization, is widely accepted.^8,11)^ TAE is effective not only for controlling active bleeding but also for reducing tumor vascularity, which may lead to tumor shrinkage.^19)^ In some cases, embolization alone may obviate the need for surgery. However, because of the risk of rebleeding, resection is generally recommended, particularly for lesions larger than 5 cm with a persistent residual tumor.^1,14,20)^ In the present case, TAE not only successfully achieved hemostasis but also resulted in substantial tumor shrinkage from 17.0 to 12.3 cm, thereby improving the patient’s condition and facilitating a safer transition to elective surgery.

Regarding the surgical approach, open hepatectomy has traditionally been considered the standard treatment for giant tumors.^21)^ However, recent evidence has shown the feasibility and safety of laparoscopic liver resection even for large (≥5 cm) or giant (≥10 cm) tumors.^22)^ Previous studies and meta-analyses have shown that laparoscopic liver resection is associated with reduced intraoperative blood loss, lower complication rates, and a shorter hospital stay than open surgery.^23,24)^ Furthermore, recent multicenter studies have reported that even for tumors larger than 10 cm, laparoscopic liver resection provides favorable perioperative outcomes without compromising long-term survival.^21,25)^ In the present case, laparoscopic resection was successfully performed for the relatively large tumor size, and the patient was discharged without postoperative complications. Additionally, the minimally invasive approach is considered to improve cosmetic outcomes, particularly for young women with HCA.

## CONCLUSIONS

This case highlights that a giant HCA may present with life-threatening hemorrhagic and thromboembolic complications even in the absence of typical risk factors. A multidisciplinary, staged approach may allow safe and minimally invasive surgical management for patients with a giant HCA complicated by hemorrhage and thromboses.

## DECLARATIONS

### Availability of data and materials

Data will be made available upon reasonable request to the corresponding author.
